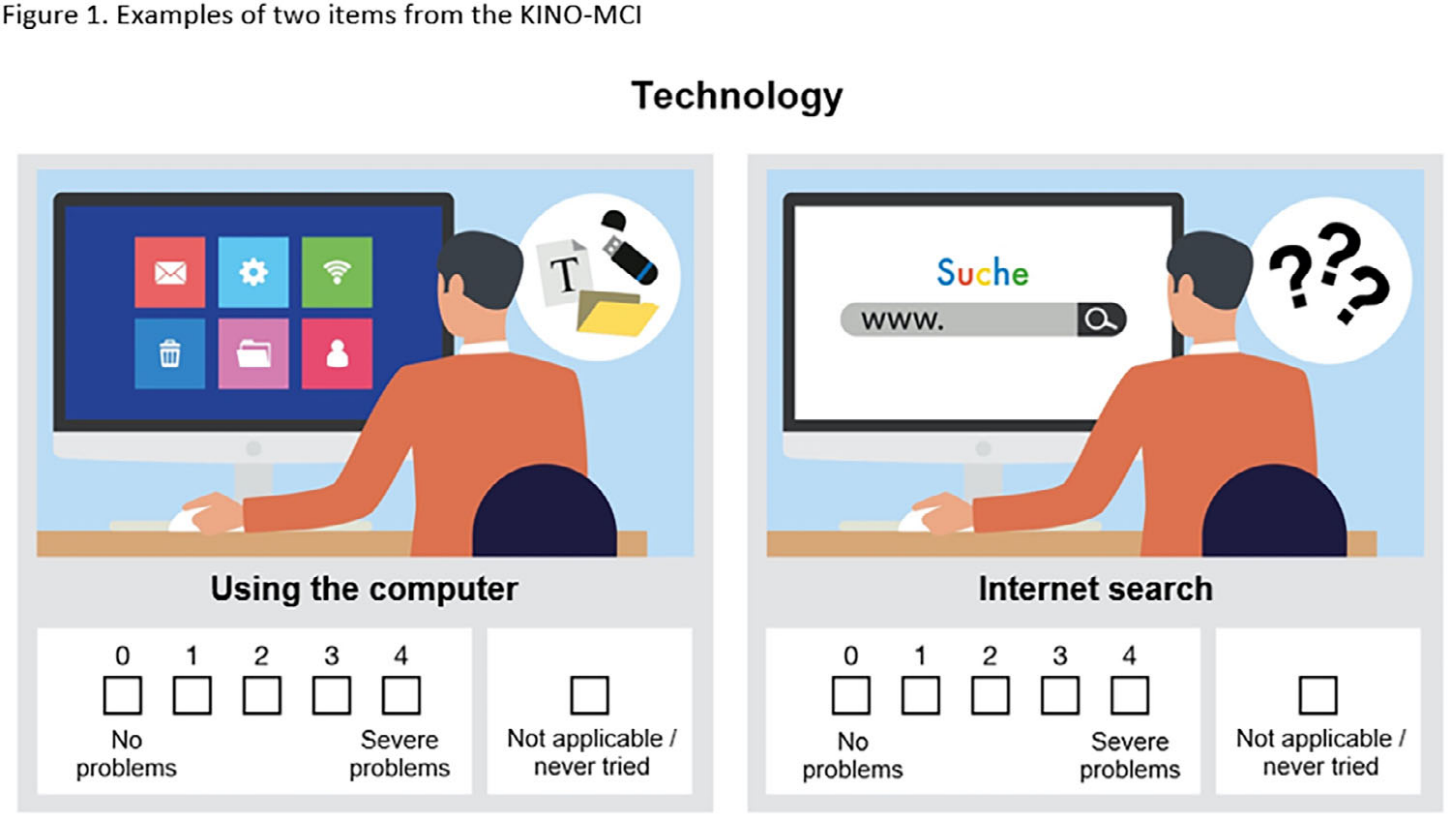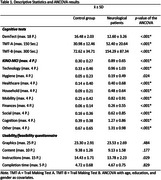# Development and validation of the Cologne instrument for the non‐verbal assessment of subjective impairments in cognition and activities of daily living (KINO‐MCI)

**DOI:** 10.1002/alz70857_101491

**Published:** 2025-12-24

**Authors:** Isabell Ballasch, Josef Kessler, Oezguer A. Onur, Stefanie T Jost

**Affiliations:** ^1^ University Hospital Cologne, Cologne, North Rhine‐Westphalia, Germany

## Abstract

**Background:**

Subjective cognitive deficits and activities of daily living (ADL) are crucial in diagnosing neurocognitive disorders. ADL remain the key criterion for differentiating mild cognitive impairment (MCI) from dementia [1]. Traditional assessment tools typically have high linguistic demands, which may not be met due to acquired language disorders, reading/writing difficulties, basic cognitive deficits, or limited language skills [2]. Furthermore, current tools are outdated and neglect digital media usage. We aimed to develop a screening questionnaire for subjective changes in cognition and ADL with minimal linguistic demands.

**Method:**

In collaboration with neuropsychologists, neurolinguists, neurologists, and a graphic designer, the picture‐based Cologne instrument for the non‐verbal assessment of subjective impairments in cognition and ADL (KINO‐MCI) was developed. It assesses subjective changes in cognition (e.g., memory) and ADL (e.g., computer use, cooking) on a 0 (no problems) to 4 (severe problems) scale (see Figure 1). Comprising 48 items across nine domains (see Table 1), it takes 10‐15 minutes to complete and targets individuals with (suspected) MCI or dementia. The study included the KINO‐MCI, a usability/feasibility questionnaire, a neuropsychological test battery, and language‐based questionnaires. Descriptive statistics, ANCOVAs (age, education, and gender as covariates), and Spearman correlations were conducted.

**Result:**

A total of 261 control subjects (women: *n* = 147(56.3%); age: x̄=56.95±8.46 years) and 45 neurological patients with subjective cognitive deficits, MCI, or dementia (women: *n* = 20(44.4%); age: x̄=68.96±9.47 years) were assessed in Cologne, Germany. Neurological patients reported significantly greater difficulties in all KINO‐MCI domains, except hygiene (see Table 1). The feedback questionnaire showed high satisfaction with graphics, content, instructions, and completion time. Regarding convergent validity, the KINO‐MCI demonstrated significant correlations with corresponding language‐based self‐report questionnaires (all *p* < .001) and external reports (all *p* < .001). The KINO‐MCI exhibits high internal consistency (Cronbach's alpha = .82) and retest reliability (.43‐.78, *p* < .001) over six weeks.

**Conclusion:**

Initial results suggest that the picture‐based KINO‐MCI is as effective as language‐based questionnaires in assessing subjective cognitive and ADL deficits. The KINO‐MCI demonstrates high reliability and validity and can assist in enhancing the diagnosis of MCI and dementia, even in individuals with language impairments or barriers.

Langa et al. (2014). *JAMA,312*(23)

Meyer et al. (2006). *Rehabil.,45*(2)